# Risk Factors for Additional Surgery after Iatrogenic Perforations due to Endoscopic Submucosal Dissection

**DOI:** 10.1155/2017/6353456

**Published:** 2017-02-20

**Authors:** Gi Jun Kim, Sung Min Park, Joon Sung Kim, Jeong Seon Ji, Byung Wook Kim, Hwang Choi

**Affiliations:** Division of Gastroenterology, Department of Internal Medicine, College of Medicine, The Catholic University of Korea, Seoul, Republic of Korea

## Abstract

*Objectives*. Endoscopic resection (ER) is commonly performed to treat gastric epithelial neoplasms and subepithelial tumors. The aim of this study was to predict the risk factors for surgery after ER-induced perforation. *Methods*. We retrospectively reviewed the data on patients who received gastric endoscopic submucosal dissection (ESD) or endoscopic mucosal resection (EMR) between January 2010 and March 2015. Patients who were confirmed to have perforation were classified into surgery and nonsurgery groups. We aimed to determine the risk factors for surgery in patients who developed iatrogenic gastric perforations. *Results*. A total of 1183 patients underwent ER. Perforation occurred in 69 (5.8%) patients, and 9 patients (0.8%) required surgery to manage the perforation. In univariate analysis, anterior location of the lesion, a subepithelial lesion, two or more postprocedure pain killers within 24 hrs, and increased heart rate within 24 hrs after the procedure were the factors related to surgery. In logistic regression analysis, the location of the lesion at the anterior wall and using two or more postprocedure pain killers within 24 hrs were risk factors for surgery. *Conclusion*. Most cases of perforations after ER can be managed conservatively. When a patient requires two or more postprocedure pain killers within 24 hrs and the lesion is located on the anterior wall, early surgery should be considered instead of conservative management.

## 1. Introduction

Endoscopic resection (ER), such as endoscopic submucosal dissection (ESD) and endoscopic mucosal resection (EMR), is widely used to treat gastric epithelial lesions and subepithelial lesions [1–5]. Compared to surgery, ER is less invasive [6–8] and is associated with better quality of life [9].

Among the various ER methods, ESD is preferred for the treatment of early gastric cancer without lymph node metastasis. An advantage of ESD is that it allows for *en bloc* and histologically complete resections [10, 11]. However, the disadvantage to ESD is that it is technically a more difficult procedure to perform and has a higher perforation rate than EMR [10, 11]. Iatrogenic perforations induced by gastric ESD and EMR have been reported to occur in approximately 4% and 1% of cases, respectively [10, 11]. Most perforations can be managed through endoscopic closure and conservative treatment, but surgery is required in rare cases [12–14]. A delay in deciding to perform surgery may cause fatal clinical outcomes in some patients. Therefore, it is important to determine whether additional surgery is required in patients with perforation after ER. Previous studies have reported several risk factors associated with gastric perforation caused by ER [15, 16]. However, no study has investigated the risk factors related to surgery in patients with iatrogenic gastric perforation by ER. The aim of this study was to predict the risk factors in patients who required surgery following ER-induced gastric perforation.

## 2. Materials and Methods

### 2.1. Patients

Patients underwent gastric EMR or ESD for gastric epithelial neoplasms or subepithelial lesions from January 2010 to March 2015, and the data were retrospectively reviewed. Patients diagnosed with ER-induced gastric perforations were included in this study. This study was approved by the Institutional Review Board of the Catholic University of Korea (OC16RISI0029).

### 2.2. Endoscopic Procedure

All endoscopic procedures were performed by five endoscopists (JSK, JSJ, BIL, BWK, and HC). ESD procedures were performed similar to the method described by Chung et al. [17]. EMR procedures were performed using two methods: EMR with circumferential precutting and EMR using a dual-channel endoscope (GIF-2T40; Olympus, Tokyo, Japan). EMR procedures were conducted in the same way as previously described [18].

### 2.3. Endoscopic and Histological Characteristics

The endoscopic characteristics of the lesions with perforation according to their location and size were retrospectively reviewed by two endoscopists (SMP and KJK). The vertical location of the lesion was classified into three regions (upper third including the fundus, cardia, and upper body; the middle third including the middle body, lower body, and angle; and the lower third including the antrum and pylorus). The circumferential location of the lesions was divided into four regions (lesser curvature, posterior wall, greater curvature, and anterior wall). The size of the lesion was defined as the longest length of the endoscopically resected specimen.

The resected specimens were mounted onto boards with pins and were fixed in 10% formalin. After fixation, the specimens were cut into 2 mm slices for histological diagnosis. The gastric epithelial lesions were classified according to the revised Vienna Classification [19]. The subepithelial lesions were diagnosed using immunohistochemical stains, including CD34, CD117, desmin, and S-100.

### 2.4. Characteristics of Procedure-Related Factors

*En bloc* resection was defined as the resection of the specimen in one piece. Complete resection was defined as the absence of remnant tumor tissue in any resected margin. Procedure time was measured using the time on the endoscopic image and was defined as the time between marking and finishing resection of the lesion.

### 2.5. Perforation

A patient was diagnosed with perforation when mesenteric fat or an intra-abdominal cavity was directly observed during the procedure (macroperforation) or when free air was observed on a plain chest X-ray or a computed tomography scan after the procedure without the endoscopist recognizing the gastric wall defect during the procedure (microperforation).

### 2.6. Management and Clinical Evaluation after the Procedure

**Figure 1:**
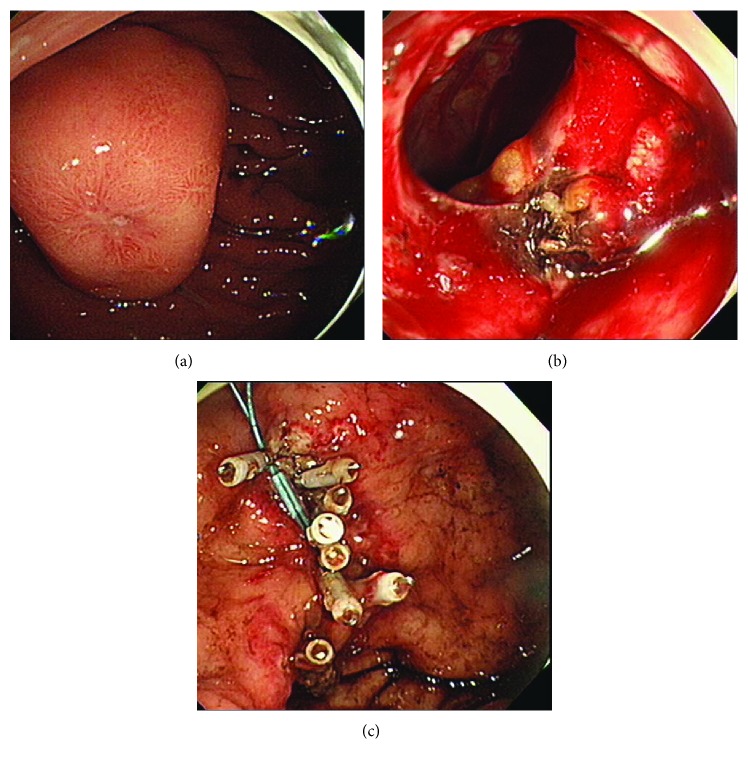
(a) A 2.8 cm GIST was observed on the anterior wall of the fundus. (b) An intra-abdominal space was detected through the perforation site during the procedure. (c) The perforation site was successfully closed using endoclips and a detachable snare.

Perforations confirmed or suspected during the endoscopic procedure were promptly closed using endoclips (HX-610-090L; Olympus, Tokyo, Japan) with or without detachable snares (MAJ 254 and MAJ 340; Olympus, Tokyo, Japan) (Figure 1). Some patients with perforation underwent percutaneous needle aspiration and received intravenous (IV) pain killers during or after the procedure to decrease their abdominal pain and were treated with antibiotics at the clinician's discretion. All patients with perforation underwent fasting until the peritoneal irritation sign and white blood cell (WBC) counts improved.

WBC counts, vital signs, physical exams, the degree of abdominal pain using a numerical rating scale, the amount of peritoneal free air, and the number of additional intravenous pain killers needed after the procedure were checked to evaluate the presence of peritonitis or sepsis. The maximum value for the white blood cell counts, heart rate, body temperature, the amount of free air, and the abdominal pain score within 24 hrs after the procedure were examined. The amount of peritoneal free air was measured using the length between the middle right diaphragm and the middle upper liver margin on a plain chest X-ray. The additional IV pain killers used after the procedure included meperidine and tramadol.

The patients underwent surgery when clinical symptoms and signs became worse depending on the judgment of the clinicians. Additional surgery was performed for EGCa that did not fulfill the extended criteria. The patients who underwent additional surgery for curative resection were classified into the nonsurgery group.

### 2.7. Statistical Analysis

Descriptive statistics were computed for all variables. Continuous data were compared using the Mann-Whitney *U* test. In univariate analysis, categorical data were compared using the *χ*^2^ test or Fisher's exact test. Multivariate analysis was performed using logistic regression analysis with backward method for variables with *P* < 0.05 in univariate analyses to confirm the risk factors related to surgery caused by perforation. Variables with *P* < 0.05 in multivariate analyses were determined as the risk factors. All analyses were performed using SPSS for Windows (version 19; SPSS Inc., Chicago, IL, USA).

## 3. Results

### 3.1. Characteristics of the Patients with Gastric Lesions and Perforations

A total of 1183 patients received gastric ER. ESD and EMR were performed in 425 and 758 patients, respectively. Perforation occurred in 69 (5.8%) patients. Among these patients, perforations occurred in 60 (14.1%) patients in the ESD group and 9 (1.2%) patients in the EMR group.

In the 69 patients, the median age was 63 (48–85) years, and 44 of the 69 patients were male (63.8%). The most common vertical location and circumferential location of the perforation were in the middle third and the lesser curvature, respectively. The median size of the resected specimen was 4.0 (0.7–14.0) cm. The pathology of the lesion included adenomas in 36 cases, EGCa in 20 cases, gastrointestinal stromal tumors in seven cases, and other types of tumors in six cases. The median procedure time was 39 (6–215) min. Macroperforation and microperforation occurred in 45 (65.2%) patients and 24 (34.8%) patients, respectively. The basic characteristics of the 69 patients and their gastric lesions are summarized in Table 1.

### 3.2. Management and Clinical Symptoms within 24 hrs

Percutaneous needle aspiration within 24 hrs after perforation was performed in nine (13.0%) patients. The median use of postprocedure pain killers was one (1–7). The median size of free air in the abdominal cavity was 2.6 (0.0–7.9) cm. The median count of white blood cells was 10,820/mm^3^ (6190–21,540), and the median degree of body temperature was 36.3 (36.0–38.2) °C. The median heart rate was 78/min (56–112).

### 3.3. Surgery

**Figure 2:**
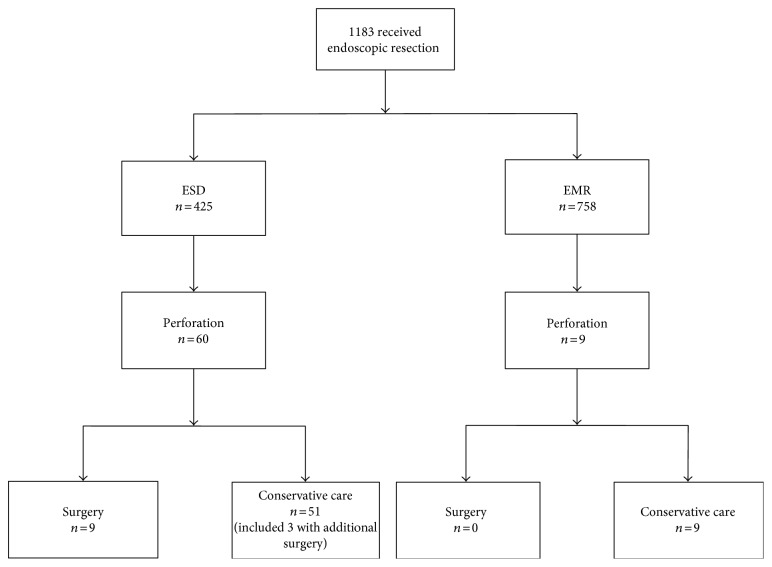
Patient selection and the clinical course of patients after gastric perforation caused by endoscopic resection. EMR, endoscopic mucosal resection; ESD, endoscopic submucosal dissection.

12 patients who received ESD underwent surgery following gastric perforation. In the EMR group, there were no patients who underwent surgery. Nine out of 12 underwent surgery because of peritonitis and sepsis caused by gastric perforation. The remaining three patients completely recovered with conservative management but received additional surgery due to noncurative resections. Therefore, these three patients were allocated into the nonsurgery group (Figure 2). Among the nine patients, six, one, and two patients received surgery on the first, second, and fifth day after the procedure, respectively. Distal gastrectomy and laparoscopic wedge resections were performed in 2 and 7 patients, respectively (Table 2). All the nine patients have fully recovered after surgery and were discharged.

### 3.4. Risk Factors for Surgery Caused by ER-Induced Gastric Perforation

In univariate analyses, the following were identified as factors associated with surgery: location of the lesion on the anterior wall (*P* = 0.000), a subepithelial lesion (*P* = 0.021), two or more postprocedure pain killers within 24 hrs (*P* = 0.000), and increased heart rate (≥100, *P* = 0.026) within 24 hrs after the procedure (Table 3). A logistic regression analysis for these four variables revealed that the location of the lesion on the anterior wall (odds ratio (OR) 20.56; 95% confidence interval (CI) 2.79–151.77, *P* = 0.003) and two or more postprocedure pain killers within 24 hrs (OR 15.13, 95% CI 2.03–112.72, *P* = 0.008) were the risk factors for surgery (Table 4).

## 4. Discussion

Gastric perforation is a serious complication associated with EMR and ESD. Although most perforations are managed through conservative treatment with endoscopic closure, surgery is needed in 2.5~3.3% of patients with perforation [12–14]. Previous studies reported that the upper area of the stomach, piecemeal resection, and long procedure times were risk factors associated with perforation after ESD [13–16, 20]. The upper area of the stomach has a thinner wall compared to other areas of the stomach and is more difficult to approach. Piecemeal resection may result from perforation that leads to irritability in patients and difficulty in procedure. Long procedure times may be due to the large size of the lesion and may be dependent on the experience of the endoscopist. Previous studies focused on the risk factors associated with the development of perforation. Most perforations can be managed conservatively with endoscopic closure. However, surgery is required in rare cases. The risk factors associated with surgery after iatrogenic colon perforation have been reported [21]. However, no study has investigated the risk factors for surgery after ER-induced gastric perforation; thus, we performed this study.

In the present study, we found that two or more postprocedure pain killers within 24 hrs was a risk factor for surgery caused by gastric perforation. The greater use of postprocedure pain killers to control pain might imply an incomplete closure of the perforation site or the continuous leakage of gastric contents through the perforation site, which will lead to peritonitis or sepsis. Therefore, surgery should be considered in patients with uncontrolled pain who do not respond to analgesics.

Additionally, the location of the lesion on the anterior wall was a risk factor for surgery in this study. In this study, 7 out of the 9 patients who underwent surgery had lesions located on the anterior wall; 6 out of the 7 patients had lesions in the upper or middle third of the stomach. Lesions on the anterior wall and in the upper and middle third appear to be in a difficult location to access and handle instruments. If a perforation occurs in that location, complete closure with endoscopic clips may be difficult. In addition, endoscopic procedures are normally performed in the lateral decubitus status, and gastric contents and blood clots may present in the upper area of the stomach due to gravity [22]. These materials can interrupt the visual field of the endoscopist and also interfere with endoscopic management when a perforation occurs. Additionally, these materials might leak through the perforation site during the procedure and induce peritonitis. Therefore, endoscopic closure should be performed earlier and more meticulously for lesions that are located on the anterior wall of the upper area of the stomach. If endoscopic closure is not performed satisfactorily, early surgery should be considered in patients with a perforation in this location.

According to a report by Cho et al. [21], the risk factors for early surgery after iatrogenic colonic perforation included large perforation (size ≥ 1 cm), leukocytosis (>10,000/mm^3^), fever ≥ 37.0°C, severe abdominal pain (narcotic pain killer use ≥ 2), and a large amount of peritoneal free air (≥3 cm). Among these risk factors, the only risk factor related to surgery after iatrogenic gastric perforation in the present study was two or more postprocedure pain killers within 24 hrs. The disparity in luminal contents may explain the difference in the risk factors for surgery after iatrogenic perforation of the colon and the stomach. Additionally, the present study included only perforations caused by ER, while the study by Cho et al. included perforations caused by diagnostic and therapeutic colonoscopy. The mechanism of perforation is different between diagnostic and therapeutic endoscopy [23, 24]. Most diagnostic perforations result from mechanical damage by the tip and shaft of the scope or a traction mechanism injury, while therapeutic perforation results from thermal injury. Therefore, diagnostic perforations are larger in size [25, 26] and need more surgical management than therapeutic perforation [24, 25]. This difference could lead to inconsistent results between the study by Cho et al. and the present study.

It is important for the attending physician to be able to determine when surgery is needed after gastric perforation caused by ER. Emergency surgery performed at night due to the deterioration of a patient's condition would be very stressful for both the surgeon and the patient. In order to avoid these worst-case scenarios, we believe, it is necessary to identify factors suggesting the need for surgery.

The present study has the following limitations: First, the present study was a retrospective study, which might lead to selection bias for the decision to perform surgery. Frequent use of pain killers may have influenced the endoscopists' decision to perform earlier surgery. In our experience, pain killers were not frequently needed when the perforation site is completely closed by clips. Thus, we believe that the need for two or more pain killers suggests inadequate closures and these patients may benefit from an earlier decision to surgery. Second, this was a single-center study with a small sample size. A multicenter study with a large sample size may be needed to apply the results of this study more generally. Finally, the perforation rate of ESD was higher than that of the previous reports and may have influenced the results.

## 5. Conclusion

In conclusion, the present study showed that the location of the lesion (on the anterior wall) and two or more postprocedure pain killers within 24 hrs were the risk factors for surgery caused by gastric perforation after ER. Therefore, early surgery for gastric perforation caused by ER should be considered in patients with these risk factors.

## Figures and Tables

**Table 1:** Basic characteristics of the 69 patients with perforation.

	Total (*n* = 69)	Nonsurgery group (*n* = 60)	Surgery group (*n* = 9)	*P* value
Age, median (range) (years)	63 (48–85)	64 (48–85)	62 (50–76)	0.695
Male sex, *n* (%)	44 (63.8)	38 (63.3)	6 (66.7)	1.000
Location (vertical), *n* (%)				0.888
Upper third	16 (23.2)	14 (23.3)	2 (22.2)	
Middle third	38 (55.1)	32 (53.3)	6 (66.7)	
Lower third	15 (21.7)	14 (23.3)	1 (11.1)	
Location (circumferential), *n* (%)				0.000
Anterior wall	14 (18.8)	7 (11.7)	7 (77.8)	
Posterior wall	18 (26.1)	18 (30.0)	0 (0.0)	
Lesser curvature	24 (34.8)	24 (40.0)	0 (0.0)	
Greater curvature	13 (18.8)	11 (18.3)	2 (22.2)	
Resected size, median (range) (cm)	4.0 (0.7–14.0)	4.0 (1.2–10.0)	4.0 (0.7–14.0)	0.748
Histologic type, *n* (%)				0.008
Adenoma	36 (52.2)	34 (56.7)	2 (22.2)	
Early gastric cancer	20 (29.0)	18 (30.0)	2 (22.2)	
Gastrointestinal stromal tumor	7 (10.2)	5 (8.3)	2 (22.2)	
Leiomyoma	1 (1.4)	1 (1.7)	0 (0.0)	
Schwannoma	2 (2.9)	0 (0.0)	2 (22.2)	
No residual tumor	3 (4.3)	2 (3.3)	1 (11.1)	
Endoscopic procedure, *n* (%)				0.594
EMR	9 (13.0)	9 (15.0)	0 (0.0)	
ESD	60 (87.0)	51 (85.0)	9 (100)	
Procedure time, median (range) (min)	39 (6–215)	38 (6–215)	39 (8–109)	0.803
*En bloc* resection, *n* (%)	62 (89.9)	53 (88.3)	9 (100)	0.582
Complete resection, *n* (%)	53 (76.8)	48 (80.0)	5 (55.6)	0.197
Perforation, *n* (%)				0.147
Macroperforation	45 (65.2)	37 (61.7)	8 (88.9)	
Microperforation	24 (34.8)	23 (38.3)	1 (11.1)	
Endoclip use, *n* (%)	55 (79.7)	46 (76.7)	9 (100)	0.187
Percutaneous needle aspiration, *n* (%)	9 (13.0)	7 (11.7)	2 (22.2)	0.333
Size of the free air in the abdominal cavity, median (range) (cm)	2.6 (0.0–7.9)	2.5 (0.0–7.9)	4.1 (0.0–7.3)	0.838
Abdominal pain score (NRS), median (range)	4 (0–10)	2.0 (0–10)	6 (4–7)	0.002
Postprocedure pain killer use, median (range)	1 (0–7)	0 (0–5)	3 (1–7)	0.000
WBC count, median (range) (mm^3^)	10,820 (6,190–21,540)	10,655 (6,190–19,140)	10,870 (9,450–21,540)	0.318
Body temperature, median (range) (°C)	36.3 (36.0–38.2)	36.3 (36.0–38.2)	36.7 (36.0–38.2)	0.149
Heart rate/min, median (range)	78 (56–112)	76 (56–112)	84 (68–110)	0.033

EMR: endoscopic mucosal resection; ESD: endoscopic submucosal dissection.

**Table 2:** Clinical characteristics, endoscopic findings, histology, surgical methods, and treatment results of patients with surgery.

Patient number	Sex	Age (years)	Tumor site (location/circumference)	Size^a^ of the resected specimen (cm)	Histology	Time of surgery after ER (day)	Cause of surgery	Surgical method	Hospital stay (day)	Results of surgery
1	M	72	Middle third/anterior	4.0	EGCa	1	Peritonitis	LWR	28	Survival
2	M	62	Lower third/anterior	15.0	Adenoma	1	Peritonitis	TLDG c Billroth I anastomosis	9	Survival
3	M	60	Middle third/anterior	7.5	Adenoma	1	Peritonitis	LWR	6	Survival
4	F	76	Middle third/anterior	5.0	Adenoma	1	Peritonitis	LWR	17	Survival
5	M	54	Upper third/anterior	1.3	GIST	1	Peritonitis	LWR	8	Survival
6	M	62	Upper third/GC	5.5	EGCa	1	Peritonitis	DSG with Billroth II anastomosis	10	Survival
7	M	69	Upper third/anterior	1.0	GIST	5	Peritonitis	LWR	4	Survival
8	F	63	Middle third/GC	0.7	Schwannoma	5	Peritonitis	LWR	19	Survival
9	F	50	Middle third/anterior	3.0	Schwannoma	2	Peritonitis	LWR	8	Survival

^a^Tumor size was determined by the measurement of the ER specimen.

DSG: distal subtotal gastrectomy; EGCa: early gastric cancer; ER: endoscopic resection; GC: greater curvature; GIST: gastrointestinal stromal tumor; LWR: laparoscopic wedge resection; TLDG: totally laparoscopic distal gastrectomy.

**Table 3:** A univariate analysis of variables associated with surgery caused by gastric perforation after endoscopic resection.

Variable	Nonsurgery group (*n* = 60)	Surgery group (*n* = 9)	*P* value
Age ≥ 70 (years)	22 (36.7)	2 (22.2)	0.480
Male sex, *n* (%)	38 (63.3)	6 (66.7)	1.000
Location (vertical), upper third, *n* (%)	13 (21.7)	3 (33.3)	1.000
Location (circumferential), anterior, *n* (%)	7 (11.7)	7 (77.8)	0.000
Resected size ≥ 3 (cm), *n* (%)	49 (81.7)	6 (66.7)	0.373
Subepithelial lesion, *n* (%)	6 (10.0)	4 (44.4)	0.021
ESD, *n* (%)	51 (85.0)	9 (100)	0.594
*En bloc* resection, *n* (%)	53 (88.3)	9 (100)	0.582
Macroperforation, *n* (%)	37 (61.7)	8 (88.9)	0.147
Size of free air in the abdominal cavity ≥ 3 (cm), *n* (%)	35 (58.3)	3 (33.3)	0.281
Abdominal pain score (NRS) ≥ 7, *n* (%)	4 (6.7)	2 (22.2)	0.172
Postprocedure pain killer use ≥ 2, *n* (%)	9 (15.0)	7 (77.8)	0.000
WBC count > 12,000/mm^3^, *n* (%)	14 (23.3)	3 (33.3)	0.679
Body temperature (>37.0°C), *n* (%)	9 (15.0)	3 (33.3)	0.183
Heart rate (≥100/min), *n* (%)	3 (5.0)	3 (33.3)	0.026

ESD: endoscopic submucosal dissection.

**Table 4:** Multivariate logistic regression analysis of risk factors for surgery caused by gastric perforation after endoscopic resection.

Variable	OR	95% CI	*P* value
Location (circumferential)	20.56	2.79–151.77	0.003
Others versus anterior			
Subepithelial lesion, *n* (%)	3.48	0.38–31.97	0.271
Postprocedure pain killer use ≥ 2, *n* (%)	15.13	2.03–112.72	0.008
Heart rate (≥100/min), *n* (%)	6.80	0.21–219.54	0.280
